# Influencing factors and coping strategies of self-management ability in patients undergoing continuous ambulatory peritoneal dialysis

**DOI:** 10.1590/1980-220X-REEUSP-2025-0214en

**Published:** 2026-02-23

**Authors:** Haiying Huang, Qiuhuang Han, Huajuan Lin, Qingfeng Chen

**Affiliations:** 1 The Third People’s Hospital of Cangnan County, Transfusion Room, 325804, Wenzhou, Zhejiang Province, China.; 2 The Third People’s Hospital of Cangnan County, Hemodialysis Room, 325804, Wenzhou, Zhejiang Province, China.; 3 The Third People’s Hospital of Cangnan County, Department of Nursing, 325804, Wenzhou, Zhejiang Province, China.; 4 The Third People’s Hospital of Cangnan County, Department of Nephrology, 325804, Wenzhou, Zhejiang Province, China.

**Keywords:** Coping Skills, Organization and Administration, Peritoneal Dialysis, Habilidades de Enfrentamento, Fator influenciador, Organização e Administração, Diálise Peritoneal

## Abstract

**Objective:**

We aimed to explore the influencing factors and coping strategies of self-management ability in patients undergoing continuous ambulatory peritoneal dialysis.

**Method:**

Stratified random sampling was employed to select 160 patients receiving continuous ambulatory peritoneal dialysis between August 2023 and October 2024. Data investigation was accomplished using the general data questionnaire, the 13-item Sense of Coherence Scale, the Self-Perceived Burden Scale, the Social Support Rating Scale, and the Self-Management Questionnaire.

**Results:**

The Self-Management Questionnaire score was (57.41 ± 4.87) points in the 160 patients. Significant differences were observed in Self-Management Questionnaire scores among patients with different ages, education levels, Self-Perceived Burden Scale scores, monthly family incomes, medical expense payment modes, 13-item Sense of Coherence scores, Social Support Rating Scale scores, and number of times they received health educational guidance received (P < 0.05). Age, education level, Self-Perceived Burden Scale score, monthly family income, medical expense payment mode, 13-item Sense of Coherence score, Social Support Rating Scale score, and number of times they received health educational guidance on received were the influencing factors of Self-Management Questionnaire scores (P < 0.05).

**Conclusion:**

Patients receiving continuous ambulatory peritoneal dialysis have medium-level self-management ability, which needs to be enhanced. Medical staff should pay attention to patients with old ages, low education levels, poor economic conditions.

## RESUMO

**Objetivo:**

Nosso objetivo foi explorar os fatores influenciadores e as estratégias de enfrentamento da capacidade de autogerenciamento em pacientes submetidos à diálise peritoneal ambulatorial contínua.

**Método:**

Foi empregada amostragem aleatória estratificada para selecionar 160 pacientes em diálise peritoneal ambulatorial contínua entre agosto de 2023 e outubro de 2024. A investigação dos dados foi realizada por meio do questionário de dados gerais, da Escala de Sentido de Coerência de 13 itens, da Escala de Carga Percebida pelo Paciente, da Escala de Avaliação de Suporte Social e do Questionário de Autogerenciamento.

**Resultados:**

A pontuação do Questionário de Autogerenciamento foi de (57,41 ± 4,87) pontos nos 160 pacientes. Diferenças significativas foram observadas nas pontuações do Questionário de Autogerenciamento entre pacientes com diferentes idades, níveis de escolaridade, pontuações na Escala de Carga Percebida, renda familiar mensal, modos de pagamento das despesas médicas, pontuações na Escala de Sentido de Coerência de 13 itens, pontuações na Escala de Avaliação de Suporte Social e número de educações em saúde recebidas (P < 0,05). Idade, nível de escolaridade, pontuação na Escala de Carga Percebida, renda familiar mensal, modo de pagamento das despesas médicas, pontuação na Escala de Sentido de Coerência de 13 itens, pontuação na Escala de Avaliação de Suporte Social e número de educações em saúde recebidas foram os fatores influenciadores das pontuações do Questionário de Autogerenciamento (P < 0,05).

**Conclusão:**

Pacientes submetidos à diálise peritoneal ambulatorial contínua possuem capacidade de autogerenciamento em nível médio, a qual necessita ser aprimorada. A equipe médica deve dar atenção a pacientes de idade avançada, baixo nível educacional e condições econômicas precárias.

## INTRODUCTION

Chronic kidney disease (CKD) is defined as damage to the structure of kidney tissues or a decline in renal function resulting from certain factors (diabetes, hypertension, and nephrotoxic drugs for example), accompanied by renal damage history ≥3 months. CKD has displayed an increasing incidence rate in recent years with the exacerbation of population aging worldwide^(1)^. In addition, it exhibits a prevalence rate of about 15% in adults, as revealed by the epidemiological survey of renal diseases in USA in 2023^(2)^. CKD is a process of worse and irreversibly progressing renal function, with end-stage renal disease (ESRD) as the final stage. To maintain life, ESRD patients often need to rely on renal replacement therapy for life.

Common regimens for renal replacement therapy in clinic include hemodialysis and peritoneal dialysis (PD). The latter includes automated peritoneal dialysis (APD) and continuous ambulatory peritoneal dialysis (CAPD). Hemodialysis must be performed in hospitals, with high costs, and APD has a high machine price and leads to more protein losses during dialysis, while CAPD possesses such advantages as zero hemodynamic changes, protection of patients’ residual function, easy operation, home treatment available, and low incidence rate of cross-infection. As a result, CAPD has become a preferred option for clinical treatment of ESRD^(3,4)^.

The concept of self-management in the field of asthma rehabilitation was first proposed in 1976 and used to describe the medical compliance and self-care ability of patients^(5)^. Afterwards, self-management was gradually introduced into the field of chronic diseases, in which self-management was defined as acquiring the ability to adapt to role and social changes, change lifestyles, and manage disease symptoms and emotions during the treatment of chronic diseases. CAPD is a process of long-term patient self-management at home, and patients are prone to negative emotions like psychological burden and laxity due to repeated dialysis operations day after day. Consequently, poor self-management and increased risks of such complications as peritonitis and exit-site infection emerge, posing negative influences on dialysis efficacy and disease outcomes^(6,7)^. In recent years, CAPD self-management has been successfully applied to digital intervention, psychosocial support, and promotion of healthy behaviors. For instance, remote monitoring and multi-dimensional health education for patients receiving automated peritoneal dialysis using technologies such as the Internet of Things and mobile applications can help improve the treatment compliance, self-management quality, and long-term prognosis^(8)^.

In view of this, the influencing factors of self-management ability in CAPD patients were analyzed in this study, and coping strategies were put forward, aiming to render theoretical support for enhancing the self-management ability in clinical practice.

## METHOD

### Subjects

This was a cross-sectional observational study. The reporting of this study followed the STROBE (Strengthening the Reporting of Observational Studies in Epidemiology) guidelines. Assuming an unknown prevalence of moderate-to-severe self-perceived burden among CAPD patients, we used the conservative estimate P = 0.50, two-sided α = 0.05 (Z_0.975_ = 1.96), and an absolute precision (margin of error) d = 0.082. Tic required sample size was: n = Z^2^ × P × (1-P)/d^2^ ≈ 143, Allowing for 10% non-response/ineligibility, the target was 159. Therefore, 160 subjects were enrolled from CAPD patients visiting our hospital from August 2023 to October 2024 through stratified random sampling. To ensure representativeness across key characteristics of the CAPD population, we used stratified random sampling based on gender (male/female), age (< 60/≥ 60 years), and dialysis duration (<12, 12–24, > 24 months), with proportional allocation to the source registry.

There were 92 males and 68 females aged 18–72 years old, with a mean of (55.41 ± 5.38) years old. The total dialysis duration ranged from 7 to 32 months, with (15.54 ± 2.12) months on average. In relation to where they lived, 25 subjects lived in urban areas and 135 subjects in rural areas. In terms of marital status, 10 were unmarried, 134 were married and 16 were either divorced or widowed.

### Ethical Aspects

This study was approved by the institutional ethics committee of The Third People’s Hospital of Cangnan County (No. TPHCC0126).

### Inclusion and Exclusion Criteria

The undermentioned inclusion criteria were adopted: Patients who met the Kidney Disease: Improving Global Outcomes (KDIGO) criteria for the diagnosis of CKD and were diagnosed with ESRD^(9)^, those who had indications for PD and received CAPD^(10)^, they also had to be able to comprehend, read and communicate normally and cooperate or answer questionnaires on their own, those who were 18–72 years old and relatively stable condition during investigation, and those signing the informed consent form at their wills.

The exclusion criteria were: Patients undergoing regular CAPD for < 3 months, those receiving renal transplantation or requiring other dialysis such as intermittent hemodialysis and intermittent APD owing to variations of the illness during investigation, those with severe CAPD complications (such as peritonitis), those with severe damage/severe infection/malignant tumors of vital organs except for kidneys (lungs, heart, brain, *etc.*), or those with physical disability/personality disorder/history of mental diseases/history of respiratory failure/history of cardiovascular or cerebrovascular accidents.

### General Data Questionnaire

A general data questionnaire was designed in accordance with study objectives, covering age (< 60 years old, or ≥ 60 years old), gender (male or female), total dialysis duration (< 12 months, 12–24 months, or > 24 months), body mass index (BMI) (< 24.0 kg/m^2^, or ≥ 24.0 kg/m^2^), educational level (primary school and below, junior high school/technical secondary school/high school, or college and above), type of residence (urban area or rural area), occupational status (on-the-job, retired, flexibly employed, or unemployed), dialysis operation (completed independently, or assisted by family members), living pattern (living alone, or living with relatives), monthly family income (< 5,000 yuan, 5,000–10,000 yuan, or > 10,000 yuan), medical expense payment modes (employee medical insurance, medical insurance for urban residents, or rural cooperative medical insurance/self-payment), marital status (unmarried, married, or divorced/widowed), primary disease [chronic glomerulonephritis (CGN), diabetic nephropathy (DN), hypertensive renal disease (HRD), or others], 24-h ultrafiltration volume (< 500 mL, 500–1000 mL, or > 1000 mL), 24-h urine volume (< 400 mL, 400–1000 mL, or > 1000 mL), number of times they received health educational guidance received (0–2 times, 3-4 times, or ≥ 5 times), quantities of dialysis bags per day (1–3 bags, 4-5 bags, or 6 bags).

### Self-Management Questionnaire (SMQ)

The Chinese version of SMQ was employed to assess the self-management ability of patients undergoing CAPD^(11)^. The questionnaire covers a total of 28 items in 5 dimensions [complication monitoring ability (4 items), abnormal situation handling ability during operation (8 items), emotion management and social return ability (4 items), diet management ability (5 items), and fluid exchange skills (7 items)]. Each item is scored 0–3 points (“never” to “always”), and the total score is 0–84 points, which is positively related to self-management ability. The Cronbach’s α coefficient of the entire questionnaire was 0.926, and the test-retest reliability r value was 0.937. The Cronbach’s α coefficient of each item was 0.650–0.908, and the test-retest reliability r value was 0.782–0.837.

### 13-Item Sense of Coherence (SOC-13) Scale

The Chinese version of SOC-13 Scale was adopted to evaluate the SOC of patients^(12)^, which consists of 13 items in three dimensions: 4 items in perception of manageability, 5 items in perception of comprehensibility, and 4 items in perception of meaning, with each item scored by Likert 7-point scale, with 1–7 points for “always no” to “often”. The total score ranges from 13 points to 91 points, with scores of 13–63 points, 64–79 points and 80–91 points indicating low, medium, and high levels of SOC, respectively. The Cronbach’s α coefficient of the entire scale was 0.76, and that of each item was 0.549–0.690.

### Self-Perceived Burden Scale (SPBS)

Self-perceived burden assessment was completed using the Chinese version of SPBS composed of a total of 10 items in three dimensions^(13)^, namely physical burden (items 1 and 2), emotional burden (items 3, 4, 5, 6, 7, 8), and financial burden (items 9 and 10). All items are scored with the Likert 5-point scale, with 1–5 points for “never” to “always”, respectively. Reverse scoring is adopted for item 8, while forward scoring is utilized for other items. The total score is 10–50 points, with scores ranging from 10–19 points (no self-perceived burden), 20–29 points (mild self-perceived burden), 30–39 points (moderate self-perceived), to 40–50 points severe (self-perceived burden). The Cronbach’s α coefficient of the entire scale was 0.874, and that of each item was 0.563–0.850.

### Social Support Rating Scale (SSRS)

The Chinese version of SSRS was employed to evaluate the social support of patients^(14)^, which covers a total of 10 items in three dimensions, namely, subjective support (items 1, 3, 4, 5), objective support (items 2, 6, 7), and utilization of support (items 8, 9, 10). Items 1–4 and 8–10 are scored using the Likert 4-point scale (with options 1, 2, 3, and 4 for 1–4 points, respectively), item 5 is scored 1–4 points for “no” to “full support”, items 6 and 7 are scored based on the number of sources, with 0 points for no source and so forth. The total score is 22–66 points, with scores of 22–32 points, 33–45 points, and 46–66 points suggesting low, medium, and high levels of social support, respectively. The Cronbach’s α coefficient of the entire scale was 0.924, and that of each item was 0.725–0.908.

### Statistical Analysis

Statistical analysis was conducted using SPSS 25.0 software. All measurement data were subjected to the normality test, and the normally distributed data were described by (‾x ± s). In the case of variance homogeneity, the independent sample t test was used for comparison between groups, and the paired sample t test was used for comparison within groups. The adjusted t test (t’ test) was used in the case of variance heterogeneity. The measurement data with skewed normal distribution were described by median (M) and interquartile range (QR), and subjected to the Mann-Whitney U test. Count data were expressed as percentages and subjected to the chi-square test. Multivariate linear regression model was utilized to identify the influencing factors of SMQ scores in patients receiving CAPD. P < 0.05 suggested that the difference was statistically significant.

## RESULTS

### SMQ Scores in Capd Patients with Different Basic Characteristics

The overall mean SMQ score among CAPD patients was 57.41±4.87 points. SMQ scores differed significantly by age, education level, SPBS, monthly family income, and medical expense payment mode (P < 0.05). Younger patients (< 60 years) had higher SMQ scores than older patients (≥ 60 years). Patients with higher education (college or above) had the best self-management ability, followed by those with secondary education, while those with primary education and below scored the lowest. SMQ scores decreased progressively with increasing SPBS, indicating that heavier perceived burden was associated with poorer self-management. Similarly, patients from families with higher monthly income showed better SMQ performance compared with those from lower income families. In terms of medical expense payment, patients covered by employee medical insurance had the highest SMQ scores, whereas those with rural cooperative insurance or self-payment had the lowest. In contrast, no significant differences in SMQ scores were found by gender, BMI, dialysis duration, place of residence, occupational status, dialysis operation, living pattern, marital status, or primary disease (all P > 0.05) (Table 1).

**Table 1:** SMQ scores in CAPD patients with different basic characteristics - Wenzhou, Zhejiang Province, China, 2023.

Item		n	SMQ score (point)	*t/F*	P
Gender	Male	92	57.38 ± 3.42	*t* = 0.132	0.895
	Female	68	57.45 ± 3.19		
Age (year)	< 60	138	58.02 ± 4.81	*t* = 4.112	<0.001
	≥ 60	22	53.58 ± 3.94		
Educational level	Primary school and below	56	55.39 ± 4.25	*F* = 16.737	<0.001
	Junior high school/technical secondary school/high school	72	57.25 ± 4.73		
	College degree or above	32	61.31 ± 5.02		
SPBS score (point)	10–19	34	60.86 ± 5.31	*F* = 24.297	<0.001
	20–29	64	58.52 ± 4.93		
	30–39	38	55.75 ± 4.52		
	40–50	24	52.19 ± 3.96		
BMI (kg/m^2^)	< 24.0	117	57.47 ± 4.08	*t* = 0.295	0.768
	≥ 24.0	43	57.26 ± 3.74		
Total dialysis duration (month)	< 12	30	58.03 ± 3.81	*F* = 1.318	0.271
	12–24	96	57.52 ± 3.74		
	> 24	34	56.55 ± 3.91		
Type of residence	Urban area	25	57.03 ± 4.13	*t* = 0.460	0.647
	Rural area	135	57.48 ± 4.56		
Occupational status	On-the-job	44	57.61 ± 4.32	*F* = 1.212	0.783
	Retired	28	57.45 ± 4.17		
	Flexibly employed	58	57.72 ± 4.28		
	Unemployed	30	56.48 ± 4.19		
Dialysis operations	Done independently	124	57.49 ± 4.72	*t* = 0.405	0.686
	Family members assist in the completion	36	57.13 ± 4.59		
Living pattern	Living alone	34	57.04 ± 4.68	*t* = 0.533	0.595
	Living with relatives	126	57.51 ± 4.53		
Monthly family income (CNY)	< 5000	16	54.27 ± 4.07	*F* = 21.233	<0.001
	5000–10000	86	55.83 ± 4.65		
	> 10000	58	60.62 ± 5.13		
Medical expense payment mode	Employee medical insurance	64	60.43 ± 5.89	*F* = 19.054	<0.001
	Medical insurance for urban residents	40	56.86 ± 5.52		
	Rural cooperative medical insurance/Self-payment	56	54.35 ± 4.75		
Marital status	Unmarried	10	57.59 ± 4.12	*F* = 0.046	0.955
	Married	134	57.43 ± 4.19		
	Divorced/widowed	16	57.13 ± 4.23		
Primary disease	CGN	34	57.61 ± 4.85	*F* = 1.045	0.752
	DN	62	57.15 ± 4.78		
	HRD	46	57.72 ± 4.91		
	Others	18	57.14 ± 4.88		

SMQ scores in CAPD patients with different characteristics.

When stratified by psychosocial characteristics and care exposure, SMQ scores varied significantly according to SOC-13 levels, SSRS levels, and number of health education sessions received (P < 0.05). Patients with higher SOC and higher social support reported higher SMQ scores, suggesting a positive association between psychosocial resources and self-management. Likewise, patients who had received health education more frequently (≥5 times) achieved the highest SMQ scores, while those with fewer sessions scored lower. In contrast, no significant differences in SMQ were observed across categories of 24-h ultrafiltration volume, 24-h urine volume, or number of dialysis bags per day (all P > 0.05) (Table 2).

**Table 2:** SMQ scores in CAPD patients with different characteristics - Wenzhou, Zhejiang Province, China, 2023.

Item		n	SMQ score (point)	*t/F*	P
24-h ultrafiltration volume (mL)	< 500	52	57.69 ± 4.12	*F* = 1.098	0.336
	500–1000	82	57.58 ± 4.23		
	> 1000	26	56.31 ± 3.97		
24-h urine volume (mL)	< 400	14	56.42 ± 3.72	*F* = 0.508	0.603
	400–1000	90	57.49 ± 3.84		
	> 1000	56	57.53 ± 3.91		
Quantities of dialysis bags per day (bags/day)	1–3	34	56.81 ± 3.85	*F* = 0.577	0.563
	4-5	114	57.54 ± 3.79		
	6	12	57.87 ± 3.82		
SOC-13 score (points)	13–63	28	50.26 ± 4.43	*F* = 32.829	<0.001
	64–79	102	58.25 ± 5.26		
	80–91	30	61.23 ± 6.74		
SSRS score (points)	22–32	32	54.56 ± 4.12	*F* = 10.326	<0.001
	33–45	106	57.68 ± 4.73		
	46–66	22	60.25 ± 4.92		
Number of times they received health educational guidance	0–2	78	55.31 ± 4.11	*F* = 21.698	<0.001
	3-4	66	58.59 ± 4.86		
	≥ 5	16	62.78 ± 5.23		

## RESULTS OF MULTIVARIATE REGRESSION ANALYSIS ON INFLUENCING FACTORS OF SMQ SCORES IN PATIENTS RECEIVING CAPD

As revealed by multivariate regression analysis, age, educational level, SPBS score, monthly family income, and number of times they received health educational guidance were the influencing factors of SMQ scores in patients undergoing CAPD (P < 0.05) (Table 3).

**Table 3:** Results of multivariate regression analysis on the influencing factors of SMQ scores in patients receiving CAPD - Wenzhou, Zhejiang Province, China, 2023.

Index	Assignment	*B*	*SE*	β	*t*	P	*95% CI*
Age	0 = <60, 2 = ≥60	-0.076	0.016	-0.469	-4.685	<0.001	-0.108~-0.044
Educational level	0 = college and above, 1 = junior high school/technical secondary school/high school, 2 = primary school and below	-0.059	0.011	-0.407	-5.607	<0.001	-0.080~-0.038
SPBS score	0 = 10–19, 1 = 20–29, 2 = 30–39, 3 = 40–50	-0.090	0.012	-0.507	-7.387	<0.001	-0.114~-0.066
Monthly family income	0 = > 10000, 1 = 5000-10000, 2 = <5000	-0.052	0.008	-0.443	-6.209	<0.001	-0.069~--0.036
Medical expense payment mode	0 = employee medical insurance, 1 = medical insurance for urban residents, 2 = rural cooperative medical insurance/self-payment	-0.064	0.010	-0.441	-6.170	<0.001	-0.084~-0.043
SOC-13 score	0 = 80–91, 1 = 64–79, 2 = 13–63	-0.048	0.006	-0.511	-7.463	<0.001	-0.061~-0.035
SSRS score	0 = 46–66, 1 = 33–45, 2 = 22–32	-0.040	0.009	-0.339	-4.531	<0.001	-0.058~-0.023
Number of times they received health educational guidance	0 = ≥5, 1 = 3-4, 2 = 0–2	-0.060	0.009	-0.464	-6.579	<0.001	-0.079~-0.042
Constant	-	4.730	0.484	-	9.772	<0.001	3.774~5.687

## DISCUSSION

In this study, multivariate regression analysis revealed that age, education level, and number of times they received health educational guidance served as the influencing factors of SMQ scores in patients undergoing CAPD, suggesting that older ages and lower education levels elevated the risk of poor self-management in patients. It is possibly attributed to the following facts.

First, older patients have gradually declined memory and reaction capacity, and the relevant precautions and operations during CAPD are relatively complicated. This finding is consistent with existing literature, where advanced age has been identified as a significant barrier to effective self-management in chronic kidney disease populations. For example, a study showed that older patients often face a confluence of challenges, including cognitive decline, polypharmacy, and physical frailty, which collectively complicate adherence to complex dialysis regimens^(15)^. An increase in age can affect the ability of patients to respond to emergencies and master fluid exchange skills during CAPD. In addition, older patients are more relaxed in daily diet management. Consequently, older patients have a low level of self-management ability. However, young patients have a relatively strong ability to understand and learn, master CAPD knowledge and related operations better and have a stronger sense of life. Hence, they can realize the importance of good self-management for slowing down the progression of ESRD, more proactively understand therapeutic regimens and detect and treat their own disease abnormalities in time, and adhere to disease management like disease monitoring and dietary structure adjustment following doctors’ advice. Accordingly, young patients have a higher level of self-management ability^(16)^.

Second, patients with low education levels have limited understanding of CAPD therapeutic regimens and daily disease management, and relatively single ways to acquire CAPD-related knowledge. This aligns with the well-established concept of health literacy, which is strongly correlated with educational attainment. Patients with limited health literacy often struggle to comprehend medical instructions, navigate the healthcare system, and make informed decisions about their care, leading to a higher risk of adverse outcomes^(17)^. Besides, there is a lack of guidance from medical staff in long-term home dialysis. As a result, these patients are more likely to have wrong cognitions and wrong behaviors. Nevertheless, patients with high education levels have relatively strong understanding and learning ability and can better understand daily disease management, correct operations of CAPD and so on, quickly master the operation of fluid exchange, and establish a correct concept of disease management. Hence, they perform better in self-management^(18)^.

Third, the number of times they received health educational guidance refer to the times they had attended health education lectures on such aspects as CAPD knowledge, operation precautions, and daily life diet management held by the community, hospitals and other organizations. Several foreign studies have manifested that continuous education and training can improve the disease knowledge reserve and self-management behavior and decrease the risk of catheter exit infection and CAPD-induced peritonitis in patients receiving CAPD^(19,20)^. This confirmes the principle that health education should not be a one-time event but a continuous process of reinforcement. Repeated engagement helps solidify knowledge and skills, ultimately fostering patient empowerment and improving clinical outcomes^(21)^. Patients who receive more health education can better grasp the standardized operation process of CAPD, and their understanding of CAPD content can be gradually enriched during multiple health education, enhancing their attention to daily disease management and participation in self-management behavior.

Moreover, the results of multivariate regression analysis uncovered that SOC and social support levels were the influencing factors of SMQ scores in patients undergoing CAPD, which could have certain impacts on the self-management ability of patients. First, SOC acts as the internal driver of patients’ adherence to self-management behavior, and its level reflects the patient’s perception of the meaning, manageability, and comprehensibility of the disease^(22)^. CAPD patients with a high SOC level can maintain a good attitude when facing the disease, give new cognition and meaning to the disease, encourage themselves to adhere to the doctor’s advice to adopt more health-friendly behaviors, accept and adapt to the role of patients and life with the disease. Accordingly, such patients maintain a good level of self-management^(23)^.

Second, it has been reported that the probability of good self-management was 2.38 times higher in ESRD patients receiving dialysis with higher social support levels than those with low social support levels, demonstrating that good social support can promote the enhancement of patient self-management ability^(24)^. This supportive network is crucial as it can buffer the psychological distress associated with a chronic illness. Strong social support has been shown to mitigate symptoms of depression and anxiety, which are known to negatively impact motivation and adherence to treatment regimens in dialysis patients^(25)^. Family care and friend visits can provide emotional support for patients undergoing CAPD and help them re-accept themselves, good social support can offer certain financial support for their continuous treatment, and scientific and reasonable disease management strategies formulated by medical staff can provide them with professional self-management information support, thereby enhancing their belief in disease management and maintaining a good level of self-management^(26,27)^.

Furthermore, as revealed by multivariate regression analysis, monthly family income, medical expense payment mode, and self-perceived burden were the influencing factors of SMQ scores in patients receiving CAPD, indicating that low monthly family incomes, medical expense payment modes of rural cooperative medical service/self-payment, and heavy self-perceived burden are not conducive to maintaining a good self-management level in patients. The financial burden of ESRD is substantial and has been increasingly recognized as a critical determinant of patient outcomes. Studies have documented that patients experiencing financial hardship are more likely to be non-adherent to medications and dietary restrictions, often forced to choose between medical necessities and other essential living expenses^(28,29)^. This is ascribed to the following facts. First, patients with low monthly family incomes or medical expense payment modes of rural cooperative medical service/self-payment have relatively poor economic situation, low or even zero insurance reimbursement ratios, and more self-paid expenses. CAPD is a lifelong long-term treatment, and patients need to not only regularly monitor the blood potassium, blood creatinine and other biochemical indicators, but also take lipid-lowering, phosphorus-lowering and other drugs for a long time. Accordingly, related costs may exceed the affordability of family economy. As a result, these patients have great economic concerns and thus easily reduce the frequencies of CAPD, re-examinations and other behaviors without authorization, and have a low level of self-management.

Second, during the long-term CAPD, patients have more worries. For instance, they worry that they bring heavy economic pressure to their families, and that the physical and mental conditions of caregivers may also be affected to varying degrees in the process of rendering long-term and complicated care to patients since caregivers need to shoulder more family care responsibilities in addition to completing their own work. The combination of various factors makes patients have a heavy self-perceived burden, making them spiritless, anxious or depressed in the treatment of the disease, and thus adopt a negative attitude toward self-management^(30)^. This psychological distress, often termed illness burden, can lead to treatment fatigue and a sense of hopelessness, directly undermining a patient’s capacity for proactive self-management^(31)^.

The implications for clinical practice are clear: a tailored, multi-pronged strategy is essential. Interventions should focus on overcoming barriers related to health literacy for older and less educated patients through simplified, multi-format education. Psychosocial support, including family involvement, peer networks, and psychological counseling, is needed to enhance patients’ sense of coherence and social resources. Finally, addressing economic hardships through financial counseling and connecting patients to social services is critical for sustainable self-management. Such a holistic approach can effectively improve self-management outcomes in this vulnerable population^(32)^.

Nevertheless, this study still has some limitations. CAPD patients were selected from only one hospital in one region using convenience sampling, which cannot represent the CAPD population in whole China, and the results may be affected by regional limitations, sampling bias, and small sample size. In addition, the factors introduced were incomplete, and other potential unexplored factors may influence the self-management ability of patients undergoing CAPD. Therefore, studies involving different regions on large samples should be carried out in the future, and whether clinical coping strategies can enhance the self-management ability of patients receiving CAPD should be further verified in clinical practice.

## CONCLUSION

The self-management ability of patients receiving CAPD is at a medium level, requiring further enhancement. Medical staff should pay attention to high-risk patients, namely patients with old ages, low education levels, poor economic conditions, and heavy psychological burdens, and promote the improvement of their self-management ability by taking reasonable intervention measures *as per* their actual situation.

## DATA AVAILABILITY

The entire dataset supporting the results of this study is available upon request to the corresponding author.
