# Supplementary material for: Influencing factors and coping strategies of self-management ability in patients undergoing continuous ambulatory peritoneal dialysis
Source: Rev Esc Enferm USP. 2026 Feb 23;60:e20250214. doi: 10.1590/1980-220X-REEUSP-2025-0214en (PMC12935461; doi:10.1590/1980-220X-REEUSP-2025-0214en)
Supplement: Translation

# Translation: Factores influyentes y estrategias de afrontamiento de la capacidad de autocontrol en pacientes sometidos a diálisis peritoneal ambulatoria continua

**DOI:** 10.1590/1980-220X-REEUSP-2025-0214es

**Authors:** Haiying Huang, Qiuhuang Han, Huajuan Lin, Qingfeng Chen

**Affiliations:** 1 The Third People’s Hospital of Cangnan County, Transfusion Room, 325804, Wenzhou, Zhejiang Province, China.; 2 The Third People’s Hospital of Cangnan County, Hemodialysis Room, 325804, Wenzhou, Zhejiang Province, China.; 3 The Third People’s Hospital of Cangnan County, Department of Nursing, 325804, Wenzhou, Zhejiang Province, China.; 4 The Third People’s Hospital of Cangnan County, Department of Nephrology, 325804, Wenzhou, Zhejiang Province, China.

**Keywords:** Habilidades de Afrontamiento, Factor influyente, Organización y Administración, Diálisis Peritoneal

## Abstract

**Objetivo:**

Nuestro objetivo fue explorar los factores influyentes y las estrategias de afrontamiento de la capacidad de autogestión en pacientes sometidos a diálisis peritoneal ambulatoria continua.

**Método:**

Se empleó un muestreo aleatorio estratificado para seleccionar a 160 pacientes en diálisis peritoneal ambulatoria continua entre agosto de 2023 y octubre de 2024. La recolección de datos se realizó mediante un cuestionario de datos generales, la Escala de Sentido de Coherencia de 13 ítems, la Escala de Carga Percibida por el Paciente, la Escala de Evaluación de Apoyo Social y el Cuestionario de Autogestión.

**Resultados:**

La puntuación del Cuestionario de Autogestión fue de (57,41 ± 4,87) puntos en los 160 pacientes. Se observaron diferencias significativas en las puntuaciones del Cuestionario de Autogestión entre pacientes con diferentes edades, niveles de escolaridad, puntuaciones en la Escala de Carga Percibida, ingresos familiares mensuales, modalidades de pago de los gastos médicos, puntuaciones en la Escala de Sentido de Coherencia de 13 ítems, puntuaciones en la Escala de Evaluación de Apoyo Social y número de actividades de educación en salud recibidas (P < 0,05). La edad, el nivel de escolaridad, la puntuación en la Escala de Carga Percibida, el ingreso familiar mensual, la modalidad de pago de los gastos médicos, la puntuación en la Escala de Sentido de Coherencia de 13 ítems, la puntuación en la Escala de Evaluación de Apoyo Social y el número de actividades de educación en salud recibidas fueron los factores influyentes en las puntuaciones del Cuestionario de Autogestión (P < 0,05).

**Conclusión:**

Los pacientes sometidos a diálisis peritoneal ambulatoria continua presentan una capacidad de autogestión de nivel medio, que necesita ser mejorada. El equipo médico debe prestar atención a los pacientes de edad avanzada, con bajo nivel educativo y en condiciones económicas precarias.

## INTRODUCCIÓN

La enfermedad renal crónica (ERC) se define como el daño a la estructura de los tejidos renales o la disminución de la función renal como resultado de ciertos factores (por ejemplo, diabetes, hipertensión y medicamentos nefrotóxicos), acompañado de un historial de daño renal ≥ 3 meses. La ERC ha mostrado una tasa de incidencia creciente en los últimos años con el agravamiento del envejecimiento de la población en todo el mundo^(1)^. Además, presenta una tasa de prevalencia de alrededor del 15 % en adultos, según revela la encuesta epidemiológica sobre enfermedades renales realizada en EE. UU. en 2023^(2)^. La ERC es un proceso de empeoramiento e irreversible progresión de la función renal, cuya etapa final es la enfermedad renal terminal (ERT). Para mantener la vida, los pacientes con ERT a menudo necesitan depender de la terapia de reemplazo renal de por vida.

Los regímenes habituales de terapia de reemplazo renal en la clínica incluyen la hemodiálisis y la diálisis peritoneal (DP). Esta última incluye la diálisis peritoneal automatizada (DPA) y la diálisis peritoneal ambulatoria continua (DPAC). La hemodiálisis debe realizarse en hospitales, con altos costos, y la DAP tiene un alto precio de la máquina y conduce a más pérdidas de proteínas durante la diálisis, mientras que la DCAP posee ventajas tales como cero cambios hemodinámicos, protección de la función residual de los pacientes, fácil operación, tratamiento en el hogar disponible y baja tasa de incidencia de infección cruzada. Como resultado, la DCAP se ha convertido en una opción preferida para el tratamiento clínico de la ERT^(3,4)^.

El concepto de autocontrol en el campo de la rehabilitación del asma se propuso por primera vez en 1976 y se utilizó para describir el cumplimiento médico y la capacidad de autocuidado de los pacientes^(5)^. Posteriormente, el autocontrol se introdujo gradualmente en el campo de las enfermedades crónicas, en el que se definió como la adquisición de la capacidad de adaptarse a los cambios sociales y de rol, cambiar los estilos de vida y controlar los síntomas de la enfermedad y las emociones durante el tratamiento de las enfermedades crónicas. La DPAC es un proceso de autocontrol a largo plazo por parte del paciente en su domicilio, y los pacientes son propensos a emociones negativas como la carga psicológica y la laxitud debido a las repetidas operaciones de diálisis día tras día. En consecuencia, surge un autocontrol deficiente y un aumento de los riesgos de complicaciones como la peritonitis y la infección del sitio de salida, lo que influye negativamente en la eficacia de la diálisis y en los resultados de la enfermedad^(6,7)^. En los últimos años, el autocontrol de la DPAC se ha aplicado con éxito a la intervención digital, el apoyo psicosocial y la promoción de comportamientos saludables. Por ejemplo, el monitorear remoto y la educación sanitaria multidimensional para pacientes que reciben diálisis peritoneal automatizada utilizando tecnologías como el Internet de las cosas y aplicaciones móviles pueden ayudar a mejorar el cumplimiento del tratamiento, la calidad del autocontrol y el pronóstico a largo plazo^(8)^.

En vista de ello, en este estudio se analizaron los factores que influyen en la capacidad de autocontrol de los pacientes en DPAC y se propusieron estrategias de afrontamiento, con el objetivo de proporcionar apoyo teórico para mejorar la capacidad de autocontrol en la práctica clínica.

## MÉTODO

### Sujetos

Se trata de un estudio observacional transversal. La presentación de este estudio siguió las directrices STROBE (Strengthening the Reporting of Observational Studies in Epidemiology). Suponiendo una prevalencia desconocida de la carga autopercibida de moderada a grave entre los pacientes con DPAC, utilizamos la estimación conservadora P = 0,50, α bilateral = 0,05 (Z_0,975_ = 1,96) y una precisión absoluta (margen de error) d = 0,082. El tamaño de la muestra requerido fue: n = Z^2^ × P × (1-P)/d^2^ ≈143. Teniendo en cuenta un 10 % de falta de respuesta/inelegibilidad, el objetivo fue de 159. Por lo tanto, se inscribieron 160 sujetos de entre los pacientes con DPAC que acudieron a nuestro hospital entre agosto de 2023 y octubre de 2024 mediante un muestreo aleatorio estratificado. Para garantizar la representatividad de las características clave de la población con DPAC, utilizamos un muestreo aleatorio estratificado basado en el sexo (hombre/mujer), la edad (< 60/≥ 60 años) y la duración de la diálisis (< 12, 12–24, > 24 meses), con una asignación proporcional al registro de origen.

Había 92 hombres y 68 mujeres de entre 18 y 72 años, con una media de (55,41 ± 5,38) años. La duración total de la diálisis osciló entre 7 y 32 meses, con una media de (15,54 ± 2,12) meses. En cuanto al lugar de residencia, 25 sujetos vivían en zonas urbanas y 135 en zonas rurales. En cuanto al estado civil, 10 eran solteros, 134 estaban casados y 16 eran divorciados o viudos.

### Aspectos Éticos

Este estudio fue aprobado por el comité de ética institucional del Tercer Hospital Popular del condado de Cangnan (n.º TPHCC0126).

### Criterios de Inclusión y Exclusión

Se adoptaron los criterios de inclusión que se mencionan a continuación: Pacientes que cumplían los criterios de la Kidney Disease: Mejora de los Resultados Globales (KDIGO) para el diagnóstico de ERC y fueron diagnosticados con ERT^(9)^, aquellos que tenían indicaciones para DP y recibieron DPAC^(10)^, también debían ser capaces de comprender, leer y comunicarse con normalidad y cooperar o responder a los cuestionarios por sí mismos, aquellos que tenían entre 18 y 72 años y un estado relativamente estable durante la investigación, y aquellos que firmaron el formulario de consentimiento informado por voluntad propia.

Los criterios de exclusión fueron: pacientes sometidos a DPAC regular durante menos de 3 meses, aquellos que recibieron un trasplante renal o que requirieron otro tipo de diálisis, como hemodiálisis intermitente y DAP intermitente, debido a variaciones de la enfermedad durante la investigación, aquellos con complicaciones graves de DPAC (como peritonitis), aquellos con daños graves, infecciones graves o tumores malignos en órganos vitales, excepto los riñones (pulmones, corazón, cerebro, etc.), o aquellos con discapacidad física, trastornos de la personalidad, antecedentes de enfermedades mentales, antecedentes de insuficiencia respiratoria, antecedentes de accidentes cardiovasculares o cerebrovasculares. *etc.*), o aquellos con discapacidad física/trastorno de la personalidad/antecedentes de enfermedades mentales/antecedentes de insuficiencia respiratoria/antecedentes de accidentes cardiovasculares o cerebrovasculares.

### Cuestionario de Datos Generales

Se diseñó un cuestionario de datos generales de acuerdo con los objetivos del estudio, que abarcaba la edad (< 60 años o ≥ 60 años), el sexo (hombre o mujer), la duración total de la diálisis (< 12 meses, 12–24 meses o >24 meses), el índice de masa corporal (IMC) (< 24,0 kg/m^2^ o ≥ 24,0 kg/m^2^), nivel educativo (escuela primaria y inferior, secundaria/escuela secundaria técnica/preparatoria o universidad y superior), tipo de residencia (zona urbana o rural), situación laboral (en activo, jubilado, empleo flexible o desempleado), operación de diálisis (realizada de forma independiente o con la ayuda de familiares), patrón de vida (viviendo solo o con familiares), ingresos familiares mensuales (<5000 yuanes, 5000–10 000 yuanes o >10 000 yuanes), modalidades de pago de los gastos médicos (seguro médico para empleados, seguro médico para residentes urbanos o seguro médico cooperativo rural/pago por cuenta propia), estado civil (soltero, casado o divorciado/viudo), enfermedad primaria [glomerulonefritis crónica (GNC), nefropatía diabética (ND), enfermedad renal hipertensiva (ERH) u otras], volumen de ultrafiltración en 24 horas (< 500 ml, 500–1000 ml o > 1000 ml), volumen de orina en 24 horas (< 400 ml, 400–1000 ml o > 1000 ml), número de veces que recibieron orientación educativa sobre salud (0–2 veces, 3-4 veces o ≥ 5 veces), cantidad de bolsas de diálisis por día (1–3 bolsas, 4-5 bolsas o 6 bolsas).

## CUESTIONARIO DE AUTOCONTROL (SELF-MANAGEMENT QUESTIONNAIRE-SMQ)

Se utilizó la versión china del SMQ para evaluar la capacidad de autocontrol de los pacientes sometidos a DPAC^(11)^. El cuestionario abarca un total de 28 ítems en 5 dimensiones [capacidad de monitorear complicaciones (4 ítems), capacidad de manejar situaciones anormales durante la operación (8 ítems), capacidad de controlar las emociones y de retorno social (4 ítems), capacidad de controlar la dieta (5 ítems) y habilidades de intercambio de líquidos (7 ítems)]. Cada ítem se puntúa de 0 a 3 puntos («nunca» a «siempre»), y la puntuación total es de 0 a 84 puntos, lo que se relaciona positivamente con la capacidad de autocontrol. El coeficiente α de Cronbach de todo el cuestionario fue de 0,926, y el valor r de fiabilidad test-retest fue de 0,937. El coeficiente α de Cronbach de cada ítem fue de 0,650–0,908, y el valor r de fiabilidad test-retest fue de 0,782–0,837.

## ESCALA DE SENTIDO DE COHERENCIA (SENSE OF COHERENCE-SOC-13) DE 13 ÍTEMS

Se adoptó la versión china de la escala SOC-13 para evaluar el SOC de los pacientes^(12)^, que consta de 13 ítems en tres dimensiones: 4 ítems en percepción de manejabilidad, 5 ítems en percepción de comprensibilidad y 4 ítems en percepción de significado, con cada ítem puntuado por una escala Likert de 7 puntos, con 1–7 puntos para «siempre no» a «a menudo». La puntuación total oscila entre 13 y 91 puntos, y las puntuaciones de 13–63 puntos, 64–79 puntos y 80–91 puntos indican niveles bajos, medios y altos de SOC, respectivamente. El coeficiente α de Cronbach de toda la escala fue de 0,76, y el de cada ítem fue de 0,549–0,690.

## ESCALA DE CARGA AUTOPERCIBIDA (SELFPERCEIVED BURDEN SCALE - SPBS)

La evaluación de la carga autopercibida se completó utilizando la versión china de la SPBS, compuesta por un total de 10 ítems en tres dimensiones^(13)^, a saber, carga física (ítems 1 y 2), carga emocional (ítems 3, 4, 5, 6, 7, 8) y carga financiera (ítems 9 y 10). Todos los ítems se puntúan con la escala Likert de 5 puntos, con 1–5 puntos para «nunca» a «siempre», respectivamente. Se adopta una puntuación inversa para el ítem 8, mientras que para los demás ítems se utiliza una puntuación directa. La puntuación total es de 10 a 50 puntos, con puntuaciones que van de 10 a 19 puntos (sin carga autopercibida), de 20 a 29 puntos (carga autopercibida leve), de 30 a 39 puntos (carga autopercibida moderada) y de 40 a 50 puntos (carga autopercibida grave). El coeficiente α de Cronbach de toda la escala fue de 0,874, y el de cada ítem fue de 0,563–0,850.

## ESCALA DE VALORACIÓN DEL APOYO SOCIAL (SOCIAL SUPPORT RATING SCALE - SSRS)

Se utilizó la versión china de la SSRS para evaluar el apoyo social de los pacientes^(14)^, que abarca un total de 10 ítems en tres dimensiones, a saber, apoyo subjetivo (ítems 1, 3, 4 y 5), apoyo objetivo (ítems 2, 6 y 7) y utilización del apoyo (ítems 8, 9 y 10). Los ítems 1–4 y 8–10 se puntúan utilizando la escala Likert de 4 puntos (con opciones 1, 2, 3 y 4 para 1–4 puntos, respectivamente), el ítem 5 se puntúa de 1 a 4 puntos para «no» a «apoyo total», los ítems 6 y 7 se puntúan en función del número de fuentes, con 0 puntos para ninguna fuente, y así sucesivamente. La puntuación total es de 22 a 66 puntos, y las puntuaciones de 22 a 32 puntos, 33 a 45 puntos y 46 a 66 puntos sugieren niveles bajos, medios y altos de apoyo social, respectivamente. El coeficiente α de Cronbach de toda la escala fue de 0,924, y el de cada ítem fue de 0,725 a 0,908.

### Análisis Estadístico

El análisis estadístico se realizó con el software SPSS 25.0. Todos los datos de medición se sometieron a la prueba de normalidad, y los datos distribuidos normalmente se describieron mediante (`x ± s). En el caso de homogeneidad de la varianza, se utilizó la prueba t de muestras independientes para la comparación entre grupos, y la prueba t de muestras emparejadas para la comparación dentro de los grupos. Se utilizó la prueba t ajustada (prueba t’) en el caso de heterogeneidad de la varianza.

Los datos de medición con distribución normal sesgada se describieron mediante la mediana (M) y el rango intercuartílico (Quartilic Range - QR), y se sometieron a la prueba U de Mann-Whitney. Los datos de recuento se expresaron en porcentajes y se sometieron a la prueba de chi-cuadrado. Se utilizó un modelo de regresión lineal multivariante para identificar los factores que influyen en las puntuaciones SMQ en pacientes que reciben DPAC. Un valor de p < 0,05 indicaba que la diferencia era estadísticamente significativa.

## RESULTADOS

### Puntuaciones Smq En Pacientes Con Dpac Con Diferentes Características Básicas

La puntuación SMQ media global entre los pacientes con DPAC fue de 57,41 ± 4,87 puntos. Las puntuaciones SMQ difirieron significativamente según la edad, el nivel educativo, el SPBS, los ingresos familiares mensuales y la forma de pago de los gastos médicos (P < 0,05). Los pacientes más jóvenes (< 60 años) obtuvieron puntuaciones SMQ más altas que los pacientes de más edad (≥ 60 años). Los pacientes con un nivel educativo más alto (universitario o superior) tenían la mejor capacidad de autocontrol, seguidos de los que tenían educación secundaria, mientras que los que tenían educación primaria o inferior obtuvieron las puntuaciones más bajas. Las puntuaciones SMQ disminuyeron progresivamente al aumentar el SPBS, lo que indica que una mayor carga percibida se asociaba con un peor autocontrol. Del mismo modo, los pacientes de familias con ingresos mensuales más altos mostraron un mejor rendimiento SMQ en comparación con los de familias con ingresos más bajos. En cuanto al pago de los gastos de salud, los pacientes cubiertos por el seguro de los empleados obtuvieron las puntuaciones SMQ más altas, mientras que los que tenían un seguro cooperativo rural o pagaban de su bolsillo obtuvieron las más bajas. Por el contrario, no se encontraron diferencias significativas en las puntuaciones SMQ por género, IMC, duración de la diálisis, lugar de residencia, situación laboral, funcionamiento de la diálisis, patrón de vida, estado civil o enfermedad primaria (todos P > 0,05) (Tabla 1).

**Tabla 1:** Puntuaciones SMQ en pacientes en DPAC con diferentes características básicas, Wenzhou, provincia de Zhejiang, China, 2023.

Elemento		n	Puntuación SMQ (puntos)	*t/F*	P
Género	Masculino	92	57,38 ± 3,42	t = 0,132	0,895
	Mujer	68	57,45 ± 3,19		
Edad (años)	< 60	138	58,02 ± 4,81	t = 4,112	<0,001
	≥ 60	22	53,58 ± 3,94		
Nivel educativo	Primaria y menos	56	55,39 ± 4,25	F = 16,737	<0,001
	Secundaria/secundaria técnica/bachillerato	72	57,25 ± 4,73		
	Título universitario o superior	32	61,31 ± 5,02		
Puntuación SPBS (puntos)	10–19	34	60,86 ± 5,31	F = 24,297	<0,001
	20–29	64	58,52 ± 4,93		
	30–39	38	55,75 ± 4,52		
	40–50	24	52,19 ± 3,96		
IMC (kg/m^2^)	< 24,0	117	57,47 ± 4,08	t = 0,295	0,768
	≥ 24,0	43	57,26 ± 3,74		
Duración total de la diálisis (meses)	< 12	30	58,03 ± 3,81	F = 1,318	0,271
	12–24	96	57,52 ± 3,74		
	> 24	34	56,55 ± 3,91		
Tipo de residencia	Área urbana	25	57,03 ± 4,13	t = 0,460	0,647
	Área rural	135	57,48 ± 4,56		
Situación laboral	En activo	44	57,61 ± 4,32	F = 1,212	0,783
	Jubilado	28	57,45 ± 4,17		
	Empleado flexible	58	57,72 ± 4,28		
	Desempleado	30	56,48 ± 4,19		
Operaciones de diálisis	Realizadas de forma independiente	124	57,49 ± 4,72	t = 0,405	0,686
	Los familiares ayudan a completarlo	36	57,13 ± 4,59		
Patrón de vida	Vive solo	34	57,04 ± 4,68	t = 0,533	0,595
	Vive con familiares	126	57,51 ± 4,53		
Ingresos familiares mensuales (CNY)	< 5000	16	54,27 ± 4,07	F = 21,233	<0,001
	5000–10000	86	55,83 ± 4,65		
	> 10000	58	60,62 ± 5,13		
Modalidad de pago de gastos médicos	Seguro médico para empleados	64	60,43 ± 5,89	F = 19,054	<0,001
	Seguro médico para residentes urbanos	40	56,86 ± 5,52		
	Seguro médico cooperativo rural/Pago por cuenta propia	56	54,35 ± 4,75		
Estado civil	Soltero	10	57,59 ± 4,12	F = 0,046	0,955
	Casado	134	57,43 ± 4,19		
	Divorciado/viudo	16	57,13 ± 4,23		
Enfermedad primaria	CGN	34	57,61 ± 4,85	F = 1,045	0,752
	DN	62	57,15 ± 4,78		
	HRD	46	57,72 ± 4,91		
	Otros	18	57,14 ± 4,88		

Puntuaciones SMQ en pacientes en DPAC con diferentes características.

Cuando se estratificaron por características psicosociales y exposición a la atención, las puntuaciones SMQ variaron significativamente según los niveles SOC-13, los niveles SSRS y el número de sesiones de educación sanitaria recibidas (P < 0,05). Los pacientes con un SOC más alto y un mayor apoyo social obtuvieron puntuaciones SMQ más altas, lo que sugiere una asociación positiva entre los recursos psicosociales y el autocontrol. Del mismo modo, los pacientes que habían recibido educación sanitaria con mayor frecuencia (≥5 veces) obtuvieron las puntuaciones SMQ más altas, mientras que los que habían recibido menos sesiones obtuvieron puntuaciones más bajas. Por el contrario, no se observaron diferencias significativas en el SMQ entre las categorías de volumen de ultrafiltración en 24 horas, volumen de orina en 24 horas o número de bolsas de diálisis al día (todas P > 0,05) (Tabla 2).

**Tabla 2:** Puntuaciones SMQ en pacientes en DPAC con diferentes características, Wenzhou, provincia de Zhejiang, China, 2023.

Elemento		n	Puntuación SMQ (puntos)	*t/F*	P
Volumen de ultrafiltración en 24 horas (ml)	< 500	52	57,69 ± 4,12	F = 1,098	0,336
	500–1000	82	57,58 ± 4,23		
	> 1000	26	56,31 ± 3,97		
Volumen de orina en 24 horas (ml)	< 400	14	56,42 ± 3,72	F = 0,508	0,603
	400–1000	90	57,49 ± 3,84		
	> 1000	56	57,53 ± 3,91		
Cantidad de bolsas de diálisis por día (bolsas/día)	1–3	34	56,81 ± 3,85	F = 0,577	0,563
	4-5	114	57,54 ± 3,79		
	6	12	57,87 ± 3,82		
Puntuación SOC-13 (puntos)	13–63	28	50,26 ± 4,43	F = 32,829	<0,001
	64–79	102	58,25 ± 5,26		
	80–91	30	61,23 ± 6,74		
Puntuación SSRS (puntos)	22–32	32	54,56 ± 4,12	F = 10,326	<0,001
	33–45	106	57,68 ± 4,73		
	46–66	22	60,25 ± 4,92		
Número de veces que recibieron orientación educativa sobre salud	0–2	78	55,31 ± 4,11	F = 21,698	<0,001
	3-4	66	58,59 ± 4,86		
	≥ 5	16	62,78 ± 5,23		

## RESULTADOS DEL ANÁLISIS DE REGRESIÓN MULTIVARIANTE SOBRE LOS FACTORES QUE INFLUYEN EN LAS PUNTUACIONES SMQ EN PACIENTES SOMETIDOS A DPAC

Según reveló el análisis de regresión multivariante, la edad, el nivel educativo, la puntuación SSRS, los ingresos familiares mensuales y el número de veces que recibieron orientación educativa sobre salud fueron los factores que influyeron en las puntuaciones SMQ en pacientes sometidos a DPAC (P < 0,05) (Tabla 3).

**Tabla 3:** Resultados del análisis de regresión multivariante sobre los factores que influyen en las puntuaciones SMQ en pacientes sometidos a DPAC - Wenzhou, provincia de Zhejiang, China, 2023.

Índice	Asignación	*B*	*SE*	β	*t*	P	*IC del 95 %*
Edad	0 = <60, 2 = ≥60	-0,076	0,016	-0,469	-4,685	<0,001	-0,108~-0,044
Nivel educativo	0 = universidad y superior, 1 = secundaria/escuela secundaria técnica/bachillerato, 2 = primaria e inferior	-0,059	0,011	-0,407	-5,607	<0,001	-0,080~-0,038
Puntuación SPBS	0 = 10–19, 1 = 20–29, 2 = 30–39, 3 = 40–50	-0,090	0,012	-0,507	-7,387	<0,001	-0,114~-0,066
Ingresos familiares mensuales	0 = >10 000, 1 = 5000–10 000, 2 = <5000	-0,052	0,008	-0,443	-6,209	<0,001	-0,069~--0,036
Modalidad de pago de los gastos médicos	0 = seguro médico para empleados, 1 = seguro médico para residentes urbanos, 2 = seguro médico cooperativo rural/pago por cuenta propia	-0,064	0,010	-0,441	-6,170	<0,001	-0,084~-0,043
Puntuación SOC-13	0 = 80–91, 1 = 64–79, 2 = 13–63	-0,048	0,006	-0,511	-7,463	<0,001	-0,061~-0,035
Puntuación SSRS	0 = 46–66, 1 = 33–45, 2 = 22–32	-0,040	0,009	-0,339	-4,531	<0,001	-0,058~-0,023
Número de veces que recibieron orientación educativa sobre salud	0 = ≥5, 1 = 3-4, 2 = 0–2	-0,060	0,009	-0,464	-6,579	<0,001	-0,079~-0,042
Constante	-	4,730	0,484	-	9,772	<0,001	3,774~5,687

## DISCUSIÓN

En este estudio, el análisis de regresión multivariante reveló que la edad, el nivel educativo y el número de veces que recibieron orientación educativa sobre salud fueron factores que influyeron en las puntuaciones SMQ de los pacientes sometidos a DPAC, lo que sugiere que las edades más avanzadas y los niveles educativos más bajos elevaban el riesgo de un autocontrol deficiente en los pacientes. Esto puede atribuirse a los siguientes hechos.

En primer lugar, los pacientes de mayor edad han experimentado un deterioro gradual de la memoria y la capacidad de reacción, y las precauciones y operaciones pertinentes durante la DPAC son relativamente complicadas. Este hallazgo concuerda con la bibliografía existente, en la que se ha identificado la edad avanzada como una barrera significativa para el autocontrol eficaz en las poblaciones con enfermedad renal crónica. Por ejemplo, un estudio demostró que los pacientes de mayor edad a menudo se enfrentan a una confluencia de retos, entre los que se incluyen el deterioro cognitivo, la polifarmacia y la fragilidad física, que en conjunto complican el cumplimiento de los complejos regímenes de diálisis^(15)^. El aumento de la edad puede afectar a la capacidad de los pacientes para responder a emergencias y dominar las habilidades de intercambio de líquidos durante la DPAC. Además, los pacientes de edad avanzada son más relajados en el manejo diario de la dieta. En consecuencia, los pacientes de edad avanzada tienen un bajo nivel de capacidad de autocontrol. Sin embargo, los pacientes jóvenes tienen una capacidad relativamente fuerte para comprender y aprender, dominar mejor los conocimientos sobre la DPAC y las operaciones relacionadas, y tienen un sentido más fuerte de la vida. Por lo tanto, pueden darse cuenta de la importancia de un buen autocontrol para ralentizar la progresión de la ERT, comprender de forma más proactiva los regímenes terapéuticos y detectar y tratar a tiempo las anomalías de su propia enfermedad, y adherirse al manejo de la enfermedad, como monitorear la enfermedad y el ajuste de la estructura dietética siguiendo los consejos de los médicos. En consecuencia, los pacientes jóvenes tienen un mayor nivel de capacidad de autocontrol^(16)^.

En segundo lugar, los pacientes con bajos niveles de educación tienen una comprensión limitada de los regímenes terapéuticos de DPAC y del manejo diario de la enfermedad, y disponen de formas relativamente únicas de adquirir conocimientos relacionados con DPAC. Esto concuerda con el concepto bien establecido de alfabetización sanitaria, que está fuertemente correlacionado con el nivel educativo. Los pacientes con una alfabetización sanitaria limitada suelen tener dificultades para comprender las instrucciones médicas, navegar por el sistema de atención de salud y tomar decisiones informadas sobre su atención, lo que conlleva un mayor riesgo de resultados adversos^(17)^. Además, existe una falta de orientación por parte del personal médico en la diálisis domiciliaria a largo plazo. Como resultado, estos pacientes son más propensos a tener cogniciones y comportamientos erróneos. No obstante, los pacientes con altos niveles de educación tienen una comprensión y una capacidad de aprendizaje relativamente sólidas y pueden comprender mejor el manejo diario de la enfermedad, el funcionamiento correcto de la DPAC, etc., dominar rápidamente el funcionamiento del intercambio de fluidos y establecer un concepto correcto del manejo de la enfermedad. Por lo tanto, obtienen mejores resultados en el autocontrol^(18)^.

En tercer lugar, el número de veces que recibieron orientación educativa sobre salud se refiere al número de veces que asistieron a conferencias de educación sanitaria sobre aspectos como los conocimientos sobre la DPAC, las precauciones de funcionamiento y el manejo de la dieta diaria, organizadas por la comunidad, los hospitales y otras organizaciones. Varios estudios extranjeros han demostrado que la educación y la formación continuas pueden mejorar los conocimientos sobre la enfermedad y el comportamiento de autocontrol, y reducir el riesgo de infección en la salida del catéter y de peritonitis inducida por la DPAC en los pacientes que reciben DPAC^(19,20)^. Esto confirma el principio de que la educación sanitaria no debe ser un evento puntual, sino un proceso continuo de refuerzo. La participación repetida ayuda a consolidar los conocimientos y las habilidades, lo que en última instancia fomenta el empoderamiento del paciente y mejora los resultados clínicos^(21)^. Los pacientes que reciben más educación sanitaria pueden comprender mejor el proceso operativo estandarizado de la DPAC, y su comprensión del contenido de la DPAC puede enriquecerse gradualmente durante la educación sanitaria múltiple, lo que mejora su atención al manejo diario de la enfermedad y su participación en el comportamiento de autocontrol.

Además, los resultados del análisis de regresión multivariante revelaron que los niveles de SOC y de apoyo social eran los factores que influían en las puntuaciones SMQ de los pacientes sometidos a DPAC, lo que podría tener ciertos impactos en la capacidad de autocontrol de los pacientes. En primer lugar, el SOC actúa como motor interno de la adherencia de los pacientes al comportamiento de autocontrol, y su nivel refleja la percepción del paciente sobre el significado, la manejabilidad y la comprensibilidad de la enfermedad^(22)^. Los pacientes con DPAC con un alto nivel de SOC pueden mantener una buena actitud al enfrentarse a la enfermedad, darle un nuevo significado y cognición, animarse a sí mismos a seguir los consejos del médico para adoptar comportamientos más saludables, aceptar y adaptarse al papel de pacientes y a la vida con la enfermedad. En consecuencia, estos pacientes mantienen un buen nivel de autocontrol^(23)^.

En segundo lugar, se ha informado de que la probabilidad de un buen autocontrol era 2,38 veces mayor en los pacientes con ERT que recibían diálisis con niveles de apoyo social más altos que en aquellos con niveles de apoyo social bajos, lo que demuestra que un buen apoyo social puede promover la mejora de la capacidad de autocontrol del paciente^(24)^. Esta red de apoyo es crucial, ya que puede amortiguar el malestar psicológico asociado a una enfermedad crónica. Se ha demostrado que un fuerte apoyo social mitiga los síntomas de depresión y ansiedad, que se sabe que afectan negativamente a la motivación y al cumplimiento de los regímenes de tratamiento en los pacientes en diálisis^(25)^. El cuidado familiar y las visitas de amigos pueden proporcionar apoyo emocional a los pacientes sometidos a DPAC y ayudarles a aceptarse de nuevo a sí mismos, un buen apoyo social puede ofrecer cierto apoyo financiero para su tratamiento continuo, y las estrategias científicas y razonables de manejo de la enfermedad formuladas por el personal médico pueden proporcionarles apoyo profesional con información sobre el autocontrol, lo que mejora su confianza en el manejo de la enfermedad y mantiene un buen nivel de autocontrol^(26,27)^.

Además, como reveló el análisis de regresión multivariante, los ingresos familiares mensuales, la forma de pago de los gastos médicos y la carga auto percibida fueron los factores que influyeron en las puntuaciones del SMQ en los pacientes que recibían DPAC, lo que indica que los bajos ingresos familiares mensuales, las formas de pago de los gastos médicos de los servicios médicos cooperativos rurales/pago por cuenta propia y la pesada carga auto percibida no favorecen el mantenimiento de un buen nivel de autocontrol en los pacientes. La carga financiera de la ERT es considerable y se reconoce cada vez más como un factor determinante de los resultados de los pacientes. Los estudios han documentado que los pacientes con dificultades económicas son más propensos a no cumplir con la medicación y las restricciones dietéticas, y a menudo se ven obligados a elegir entre las necesidades médicas y otros gastos básicos^(28,29)^. Esto se atribuye a los siguientes hechos. En primer lugar, los pacientes con bajos ingresos familiares mensuales o con modalidades de pago de gastos médicos de servicio médico cooperativo rural/pago por cuenta propia tienen una situación económica relativamente precaria, índices de reembolso del seguro bajos o incluso nulos, y más gastos a su cargo. La DPAC es un tratamiento a largo plazo que dura toda la vida, y los pacientes no solo deben monitorear regularmente el potasio en sangre, la creatinina en sangre y otros indicadores bioquímicos, sino que también deben tomar medicamentos hipolipemiantes, hipofosfatémicos y otros durante mucho tiempo. En consecuencia, los costos relacionados pueden superar la capacidad económica de la familia. Como resultado, estos pacientes tienen grandes preocupaciones económicas y, por lo tanto, tienden a reducir la frecuencia dla DPAC, las revisiones médicas y otras conductas sin autorización, y tienen un bajo nivel de autocontrol.

En segundo lugar, durante la DPAC a largo plazo, los pacientes tienen más preocupaciones. Por ejemplo, les preocupa que supongan una gran presión económica para sus familias y que las condiciones físicas y mentales de los cuidadores también se vean afectadas en mayor o menor medida en el proceso de prestar cuidados prolongados y complicados a los pacientes, ya que los cuidadores deben asumir más responsabilidades familiares además de completar su propio trabajo. La combinación de diversos factores hace que los pacientes tengan una gran carga auto percibida, lo que los hace sentir desanimados, ansiosos o deprimidos en el tratamiento de la enfermedad y, por lo tanto, adoptar una actitud negativa hacia el autocontrol^(30)^. Esta angustia psicológica, a menudo denominada carga de la enfermedad, puede conducir a la fatiga del tratamiento y a una sensación de desesperanza, lo que socava directamente la capacidad del paciente para el autocontrol proactivo^(31)^.

Las implicaciones para la práctica clínica son claras: es esencial una estrategia personalizada y múltiple. Las intervenciones deben centrarse en superar las barreras relacionadas con la alfabetización sanitaria de los pacientes de edad avanzada y con menor nivel educativo mediante una educación simplificada y en múltiples formatos. Se necesita apoyo psicosocial, incluida la participación de la familia, las redes de compañeros y el asesoramiento psicológico, para mejorar el sentido de coherencia y los recursos sociales de los pacientes. Por último, abordar las dificultades económicas mediante el asesoramiento financiero y poner a los pacientes en contacto con los servicios sociales es fundamental para un autocontrol sostenible. Este enfoque holístico puede mejorar eficazmente los resultados del autocontrol en esta población vulnerable^(32)^.

No obstante, este estudio sigue teniendo algunas limitaciones. Los pacientes con DPAC fueron seleccionados de un solo hospital de una región mediante un muestreo por conveniencia, lo que no puede representar a la población con DPAC de toda China, y los resultados pueden verse afectados por limitaciones regionales, sesgos de muestreo y el pequeño tamaño de la muestra. Además, los factores introducidos eran incompletos y otros factores potenciales no explorados pueden influir en la capacidad de autocontrol de los pacientes sometidos a DPAC. Por lo tanto, en el futuro se deben llevar a cabo estudios que incluyan diferentes regiones con muestras de gran tamaño, y se debe verificar en la práctica clínica si las estrategias de afrontamiento clínico pueden mejorar la capacidad de autocontrol de los pacientes que reciben DPAC.

## CONCLUSIÓN

La capacidad de autocontrol de los pacientes que reciben DPAC se encuentra en un nivel medio, por lo que es necesario mejorarla. El personal médico debe prestar atención a los pacientes de alto riesgo, es decir, los pacientes de edad avanzada, con bajo nivel educativo, en condiciones económicas precarias y con una gran carga psicológica, y promover la mejora de su capacidad de autocontrol tomando medidas de intervención razonables en función de su situación real.

## DISPONIBILIDAD DE DATOS

El conjunto de datos que respaldan las conclusiones de este estudio está disponible previa solicitud al autor correspondiente.
